# Lectin‐Histochemical Pattern on the Cystic and Neoplastic Ovaries of Bitches

**DOI:** 10.1111/rda.70075

**Published:** 2025-05-14

**Authors:** Juliano P. Terra, Thiago B. Almeida, Lucas P. O. Rezende, Claudio G. Barbeito, Francisco Acuña, Guilherme R. Blume, Rômulo S. A. Eloi, Letícia B. Oliveira, André L. R. M. Santos, Fabiano J. F. Sant'Ana

**Affiliations:** ^1^ Laboratório de Diagnóstico Patológico Veterinário (LDPV) Universidade de Brasília (UnB) Brasília Brazil; ^2^ Facultad de Ciencias Veterinarias (FCV) Universidad Nacional de La Plata (UNLP) La Plata Argentina; ^3^ Laboratório HistoPato Análise Anatomopatológica Brasília Brazil

**Keywords:** dogs, glycoconjugates, lectins, ovarian cysts, ovarian neoplasia, reproductive pathology

## Abstract

Studies have demonstrated that many reproductive disorders can compromise the binding pattern of glycosidic residues, mainly in cows and sows. In the current study, the binding pattern of lectins was characterised and compared in normal and pathological ovaries of bitches. Fourteen biotinylated lectins (BSA‐1B, Con‐A, DBA, LCA, PHA‐E, PHA‐L, PNA, RCA‐1, SBA, SJA, STA, WGA, s‐WGA, UEA‐1) were used. In ovaries without lesions, the predominant staining for RCA‐1 was observed in the zona pellucida (ZP) of initial follicles, in the oocytes of primordial and primary follicles, and in the artery wall. For WGA, the reaction predominated in the mesothelium, in the ZP of secondary follicles, in the corpus luteum (CL) and in the oocyte of secondary follicles. Reactivity for STA was more pronounced in the mesothelium and ZP of developing follicles. The mesothelial cells and the CL were mainly marked for LCA. PHA‐L was stained in the mesothelium, CL, and connective stroma, while the lectin PHA‐E showed reactivity especially in the stroma, mesothelium, and artery wall. In the cystic ovaries, changes in the reaction of some lectins were noted in various structures adjacent to the cysts. RCA‐1 staining was reduced in the ZP and oocyte of primordial, primary, and secondary follicles, while there was an increase of staining in endothelial cells. With WGA lectin, there was a reduction in CL and an increase in endothelial cells. Reactivity to STA was reduced in the ZP of primary and secondary follicles and increased in endothelial cells. LCA was also reduced in the CL and ZP of primary and secondary follicles. For the PHA‐L lectin, the staining was reduced in the mesothelial cells, CL, and ZP of the primary follicles, while for PHA‐E the reduction occurred in the stroma, oocyte of the primary follicles, and in the GC of the secondary follicles. In all neoplasms analysed (dysgerminoma, granulosa cell tumour, adenocarcinoma), there was also a reduction in reactivity to various lectins, both in the neoplastic cells compared to the cells of origin and in the adjacent non‐neoplastic ovarian tissue. The data from the current study suggest that the changes in ovarian glycosidic residues observed in cysts and neoplasms may contribute to infertility in bitches.

## 1 Introduction

In recent decades, canine reproduction has evolved considerably due to investments in new biotechnological tools, as well as the search for solutions to infertility problems, especially in high‐value breeds. Persistent anestrus, irregular estrous cycles and abortions are some of the clinical problems observed. In some situations, systemic or local changes of endocrine, nutritional, infectious, or congenital nature may be involved and need to be investigated in the clinical‐reproductive assessment (Grundy et al. 2002; Lanna et al. 2012).

Among the causes of infertility in adult bitches ( *Canis familiaris* ), ovarian changes, particularly follicular cysts and neoplasms, represent an important percentage of reproductive impairment. These lesions often prevent ovulation and/or deregulate the estrous cycle. In addition, these injuries can represent significant economic losses when they affect high value females that are bred for commercial purposes (Marchevsky et al. 1983; Patnaik and Greenlee 1987; McEntee 1990; Malm et al. 1994; Sforna et al. 2003; Akihara et al. 2007; Knauf et al. 2014; Arlt and Haimerl 2016). In a study conducted in Brazil, in which 200 ovaries and uterine horns of female dogs were evaluated, 16.5% and 6.5% of the samples were diagnosed with ovarian cysts and neoplasms, respectively (Marchevsky 1981).

In domestic animals, cystic ovarian disease is more widely studied and known in cows ( *Bos taurus* ) and sows ( *Sus scrofa* ). The mechanisms that lead to the development of follicular cysts have been the subject of study and research for many years but are still poorly understood. The condition is believed to have a multifactorial aetiology (Peter 2004). In cows, cyst formation has been associated with many environmental, clinical, and hereditary factors (Garverick 1997; Monniaux et al. 2008). Some researchers point to endocrine imbalances involving the hypothalamic–pituitary axis related to the development of ovarian cysts in sows (Scholten and Liptrap 1978) and cows (Garverick 1997; Silvia et al. 2002; Vanholder et al. 2006). In addition, the detailed etiopathogenesis of the canine ovarian cysts is unknown (Ortega et al. 2016).

In adult bitches, the most frequent ovarian neoplasms are those of epithelial origin and granulosa cell tumours (GCT) (Patnaik and Greenlee 1987; Sforna et al. 2003). These two types of neoplasms are clinically important in female dogs. One study showed that 22.86% of 70 bitches with reproductive problems who were submitted to ovarian hysterectomy had GCT (Malm et al. 1994). The vast majority of studies on canine ovarian neoplasms detail the frequency and clinical, hormonal, ultrasonographic and/or anatomopathological aspects, generally with a limited number of samples analysed and lacking investigations related to the etiopathogenesis of these changes (Patnaik and Greenlee 1987; McEntee 1990; Sforna et al. 2003; Arlt and Haimerl 2016; Makovicky et al. 2023; Troisi et al. 2023; Dolensek et al. 2024).

Lectin histochemistry (LHC) is an important technique used very effectively to identify and characterise in situ carbohydrates that were used in ovarian follicles of many species, including women, rats (*Rattus* spp.), sows, and mares ( *Equus caballus* ) (Maymon et al. 1994; Salvetti et al. 2000; Desantis et al. 2009; Barbeito et al. 2013; Mendes et al. 2019). Lectins are a group of proteins that have the property of binding to certain specific carbohydrate chain structures, including those related to glycoproteins, glycolipids, and glycosaminoglycans (Debray et al. 1981; Desantis et al. 2009). In rats with experimentally induced polycystic ovaries, it has been shown that there are evident changes in the lectin‐histochemical pattern in different ovarian structures and that these changes are probably related to the process of cystogenesis (Barbeito et al. 2013). A very similar altered pattern of glycosidic residues was observed in the cystic ovaries of sows, compared to normal ovaries (Mendes et al. 2019).

In the canine ovary, the distribution of oligosaccharides has already been determined in the zona pellucida throughout follicular growth (Parillo and Verini‐Supplizi 1999; Parillo et al. 2005). However, the normal lectin histochemical pattern in other cyclical ovarian structures and in cystic or neoplastic lesions remains unknown. In human reproduction, some studies have shown that lectin histochemistry has been useful in the diagnosis and investigation of the pathogenesis and as potential biomarkers of ovarian neoplasms (Murakami 1991; Sasano et al. 1991; Bychkov et al. 1992; Oinam et al. 2022; Pallar et al. 2025). The aim of this study was to characterise and compare the binding pattern of lectins in cystic and neoplastic ovaries of female dogs.

## 2 Materials and Methods

### 2.1 Legal Aspects

The project was conducted in accordance with the Ethical Principles of Animal Care and Research and under the approval of the Ethics Committee on Animal Use (CEUA) of the Universidade de Brasilia, Brazil, protocol number 23106.067439/2023–34.

### 2.2 Samples

Ovaries from 25 sexually mature bitches with and without lesions were selected and used in this study: 6 without changes (control, 4 in luteal and 2 in follicular phase) and 19 with lesions: cystic corpus luteum (*n* = 3), follicular cysts (*n* = 3), granulosa cell tumours (*n* = 5), papillary adenocarcinomas (*n* = 4) and dysgerminomas (*n* = 4) (Table 1). The samples were obtained from the routine files of two veterinary diagnostic laboratories: *Laboratório de Diagnóstico Patológico Veterinário* of Universidade de Brasília (UnB) and *Laboratório Histopato Análise Anatomopatológica*, both in Brasília, Brazil. The samples were previously fixed in 10% neutral buffered formalin for 24 h, routinely processed for paraffin embedding, sectioned at 4–5 μm thickness and stained with haematoxylin–eosin (HE).

**TABLE 1:** Data of the bitches used in this study.

Group of ovaries	Bitch	Breed	Age (years)
Control—luteal phase	1	Mixed	5
	2	Mixed	4
	3	Mixed	6
	4	Boxer	8
Control—follicular phase	5	Doberman	6
	6	Mixed	5
Cystic corpus luteum	7	Shih‐Tzu	9
	8	Maltese	6
	9	Mixed	2
Follicular cysts	10	Cocker Spaniel	11
	11	French Bulldog	7
	12	Lhasa Apso	11
Granulosa cell tumours	13	French Bulldog	9
	14	Brazilian Fila	8
	15	Labrador Retriever	8
	16	German Shepherd	7
	17	Shih‐Tzu	11
Papillary adenocarcinomas	18	Weimaraner	13
	19	Yorkshire	10
	20	German Shepherd	9
	21	Mixed	12
Dysgerminomas	22	Dachshund	13
	23	Dachshund	9
	24	Mixed	8
	25	Mixed	8

### 2.3 LHC Study

After deparaffinisation, additional ovary sections were incubated in 0.3% hydrogen peroxide in methanol for 30 min at room temperature, washed several times in 0.01 M phosphate buffered saline (PBS), pH 7.2, and treated with 0.1% bovine serum albumin in PBS for 15 min. Subsequently, incubation was carried out with the 14 biotinylated lectins: GSA‐1B, Con‐A, DBA, LCA, PHA‐E, PHA‐L, PNA, RCA‐1, SBA, SJA, STA, WGA, s‐WGA, and UEA‐1 (Lectin Kit, Vector Laboratories Inc., Burlingame, CA, US) (Table 2).

**TABLE 2:** Lectins used in this study and their main specificities.

Acronym	Source	Dilution (μg/mL)	Main glycosidic specificity	Company
BSA‐1B	*Bandeiraea* (*Griffonia*) *simplicifolia*	30	Galα1, 3Gal‐; Galα1, 4Gal	Vector Laboratories
Con‐A	*Concanavalina ensiformis*	30	α‐D‐Man, α‐D‐Glc	Vector Laboratories
DBA	*Dolichos biflorus* (horse gram)	30	GalNAcα1, 3 (LFucα1, 2) Gal‐β1, 3/4GlcNAc β1‐ α‐D‐GalNAc	Vector Laboratories
LCA	*Lens culinaris*	30	α Man, α Glc	Vector Laboratories
PHA‐E	*Phaseolus vulgaris* *erythroagglutinin* (*Kidney bean*)	30	Bisected complex N‐linked sequences	Vector Laboratories
PHA‐L	*Phaseolus vulgaris* *leukoagglutinin* (*Kidney bean*)	30	β1‐6‐linked GlcNAc in tri/tetra‐antennary complex N‐linked sequences	Vector Laboratories
PNA	*Arachis hypogaea* (peanut)	10	Galβ1,3GlcNAcβ1‐ > Galβ1,4GlcNAcβ1—	Vector Laboratories
RCA‐1	*Ricinus communis*	30	β‐D‐Gal	Vector Laboratories
SBA	*Glycine max* (soybean)	30	Terminal GalNAcα1‐ > Galα1	Vector Laboratories
SJA	*Sophora japonica*	30	α e β GalNac > α e β Gal	Vector Laboratories
STA	*Solanum tuberosum*	30	(GlcNac) 2–4	Vector Laboratories
WGA	*Triticum vulgaris* (wheat germ)	30	α‐D‐GlcNAc, NeuNAcα2,3	Vector Laboratories
s‐WGA	Succinyl‐WGA	30	β1‐4‐D‐ GlcNac	Vector Laboratories
UEA‐1	*Ulex europaeus‐1* (gorse)	30	L‐Fucα1, 2Galβ1, 4‐GlcNAcβ1—	Vector Laboratories

Abbreviations: Fuc, fucose; Gal, galactose; GalNAc, N‐acetyl‐galactosamine; Glc, glucose; GlcNAc, N‐acetyl‐glucosamine; Man, mannose; NeuNAc, neuraminic acid.

The ideal concentration for each lectin, which allowed maximum staining with minimum background, was at a dilution of 30 μg/mL in PBS, except for PNA, which was applied at a concentration of 10 μg/mL for 1 h, followed by incubation with the avidin‐biotin complex peroxidase (ABC) (Vector Laboratories Inc.) for 45 min. Horseradish peroxidase was activated by incubation for 4–10 min with a buffered solution of 0.05 M Tris–HCl, pH 7.6, containing 0.02% diaminobenzidine (DAB) (DakoCytomation, Carpinteria, CA, USA) and 0.05% H_2_O_2_. All sections were counterstained with Mayer's haematoxylin. As a negative control, lectins were omitted or blocked by incubating them with their blocking carbohydrates (0.1–0.2 M in PBS) for 1 h at room temperature before applying them to the sections. As a positive control, canine samples with confirmed positive reactivity for all lectins employed were utilised (Zanuzzi et al. 2024). Lectin binding was analysed using the following semi‐quantitative scale of the stained structures and scored subjectively as follows: (−) none, (+) weakly positive, (++) moderately positive, and (+++) strongly positive. The structures evaluated were granulosa cells (GC), theca interna (TI), theca externa (TE), zona pellucida (ZP), and oocyte in normal growing and cystic follicles, granulosa in atretic follicles, mesothelium, corpus luteum (CL), connective stroma, and endothelium and vascular wall, as well as tumour cells in neoplasms. Two pathologists analysed the samples blindly the samples.

## 3 Results

**FIGURE 1:**
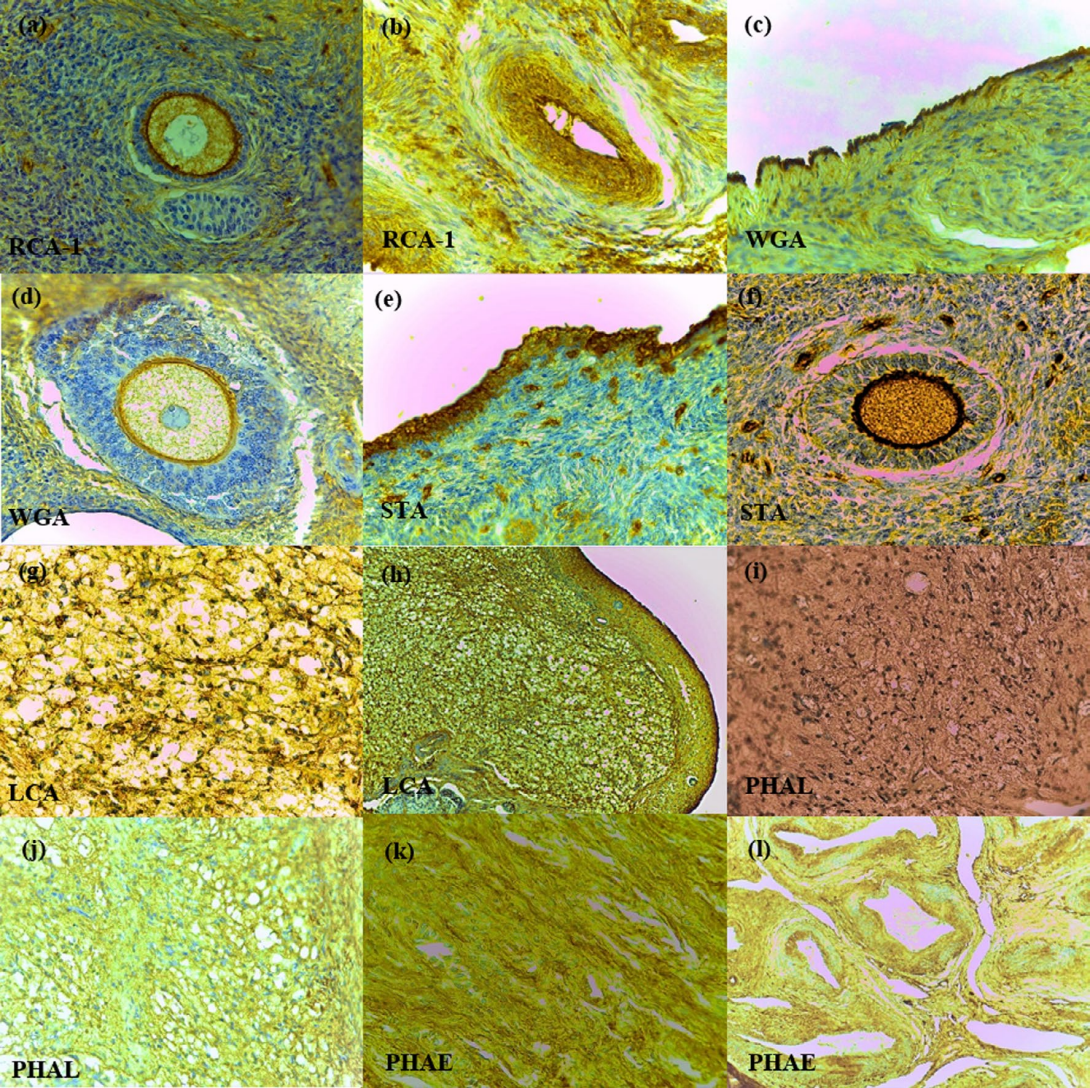
Normal ovaries. Oocyte of primary follicle (a) and media of artery (b) with moderate to strong reactivity to RCA‐1, 400×. Strong marcation to WGA in mesothelial cells (c) (200×) and moderate in oocyte of secondary follicle (d) (400×). Mesothelium (e) (200×) and zona pellucida (f) (400×) with strong reaction to STA. Corpus luteum (g) (400×) and mesothelial cells (h) (200×) present moderate to strong reactivity to LCA. Stroma (i) and corpus luteum (j) show moderate and strong reaction to PHAL, respectively, 400×. Stroma (k) and arterial wall (l) with strong marcation to PHAE, 400×.

The results of the LHC reactivity are summarised in Table 3. When there was variation in the intensity of the lectin‐histochemical reaction within the same structure between animals, these results were expressed to demonstrate the variation (Table 2). There are no LHQ changes in the normal ovaries between luteal and follicular phases. In the ovaries without lesions (Figure 1a), there was moderate to marked marcation for RCA‐1, mainly in ZP of non‐antral follicles, in the oocytes of primordial and primary follicles and in the media of arteries (Figure 1b). For WGA, the reaction was strong in the mesothelium (Figure 1c) and ZP of secondary follicles (Figure 1d), and moderate in the CL and oocyte of secondary follicles. Reactivity to STA was more pronounced in the mesothelium (Figure 1e) and ZP of developing follicles (Figure 1f). Mesothelial cells (Figure 1h) and CL (Figure 1g) showed moderate to strong reaction for LCA. PHAL showed marked positivity in the stroma (Figure 1i) and moderate in the CL and connective stroma (Figure 1j), while the lectin PHA‐E showed more prominent reactivity in the stroma (Figure 1k), mesothelium and artery wall (Figure 1l).

**TABLE 3:** Lectinohistochemical reaction in different structures of canine ovaries without lesions, cystic, and neoplastic changes.

	Con‐A	SBA	RCA‐1	DBA	PNA	WGA	UEA‐1	BSA‐1	STA	LCA	SJA	s‐WGA	PHAL	PHAE
Ovaries without lesions														
Mesothelium	−	−/+	−/+	−/+	−	+/+++	−	−	+++	++/+++	−	−	++/+++	+/++
Corpus luteum	−	−	−	−/+	−/+	+/++	−	−	+	+/++	−/+	−	+/++	−
Stroma	−	−	−/++	−	+	−/+	−	−	−/+	−/+	−/+	−/+	+/++	+/+++
Endothelium	−	−	−	−	−	−	−	−	−/+	−	−	−	−	−
Adventitia and media of arteries	−	−	−/++	−	−	−/+	−	−/++	−/++	−/+	−	−	−/+	+/++
Primordial follicles														
Granulosa cells	−	−	−/+	−	−	−	−	−	−/+	−	−	−	−	−/+
Zona pellucida	−	−	+/++	−	−	−	−	−	+++	−	−	−	−	−/+
Oocyte	−	−	−/+	−	−	+	−	−	++	−/+	−/+	−	−	+
Secondary follicles														
Thecas interna and externa	−	−	−/+	−	−	−	−	−	−	−	−	−	−	−
Granulosa cells	−	−	−	−	−	−	−	−	−	−/+	−	−	−	+
Zona pellucida	+	−	+/+++	−/+	+	+++	−/+	−	++	+	−	−	−	+/++
Oocyte	−	−	+/++	−	+	++	−	−	−	−	−	−	−	−
Tertiary follicles														
Thecas interna and externa	−	−	−	−	−	−	−	−	−	−	−	−	−	−
Granulosa cells	−	−	−	−	−	−	−	−	−/+	−	−	−	−	−
Follicular fluid	−	−	−	−	−	−	−	−	−	−	−	−	−	−
Zona pellucida	−/+	−/+	−	−	−	−	−	−	++	−/+	−	−	−	−
Oocyte	−	−	−	−	−	−	−	−	−	−	−	−	−	−
Atretic follicles														
Granulosa cells	−	−	−	−	−	−	−	−	−	−	−	−	−	−
Folicullar cyst														
Lining epithelium	−	−	−	−	−	++	−	+	−/++	−	+	−	−	−/+
Thecas interna and externa	−	−	−	−	−	−	−	−	−	−	−	−	+	−
Cystic corpus luteum														
Lining epithelium	−	−	−	−	−	−/+	−	−/+	−	−/+	−/+	−	−	−/+
Thecas interna and externa	−	−	−/+	−	−	−/+	−	−/+	−	−	−	−	−	+
Dysgerminoma														
Citoplasm of neoplastic cells	−	−	−	−	−	−	−	−	−	−	−	−/+	−/++	−/++
Citoplasmic membrane	−	−	−	−	−	−/+	−	−	−	−/+	−	−	−/+	−/++
Nucleus of neoplastic cells	−	−	−	−	−	−	−	−	−	−	−	−	−	−
Granulosa cell tumour														
Citoplasm of neoplastic cells	−	−	−/+	−	−	+	−	−	−/+	−/+	−	−/+	−/+	−/++
Citoplasmic membrane	−	−	−/+	−	−	−	−	−	−	−/+	−	−	−/+	−/++
Nucleus of neoplastic cells	−	−	−	−	−	−	−	−	−	−	−	−	−	−
Papillar adenocarcinoma														
Citoplasm of neoplastic cells	−	−	−	−	−	−	−	−	−/+	−	−	−	−	+/++
Citoplasmic membrane	−	−	−	−	−	+	−	−	−/++	−	−	−	−/+	−/++
Nucleus of neoplastic cells	−	−	−	−	−	−	−	−	−	−	−	−	−	−

*Note:* (−) none, (+) weakly positive, (++) median positive, (+++) strongly positive.

**FIGURE 2:**
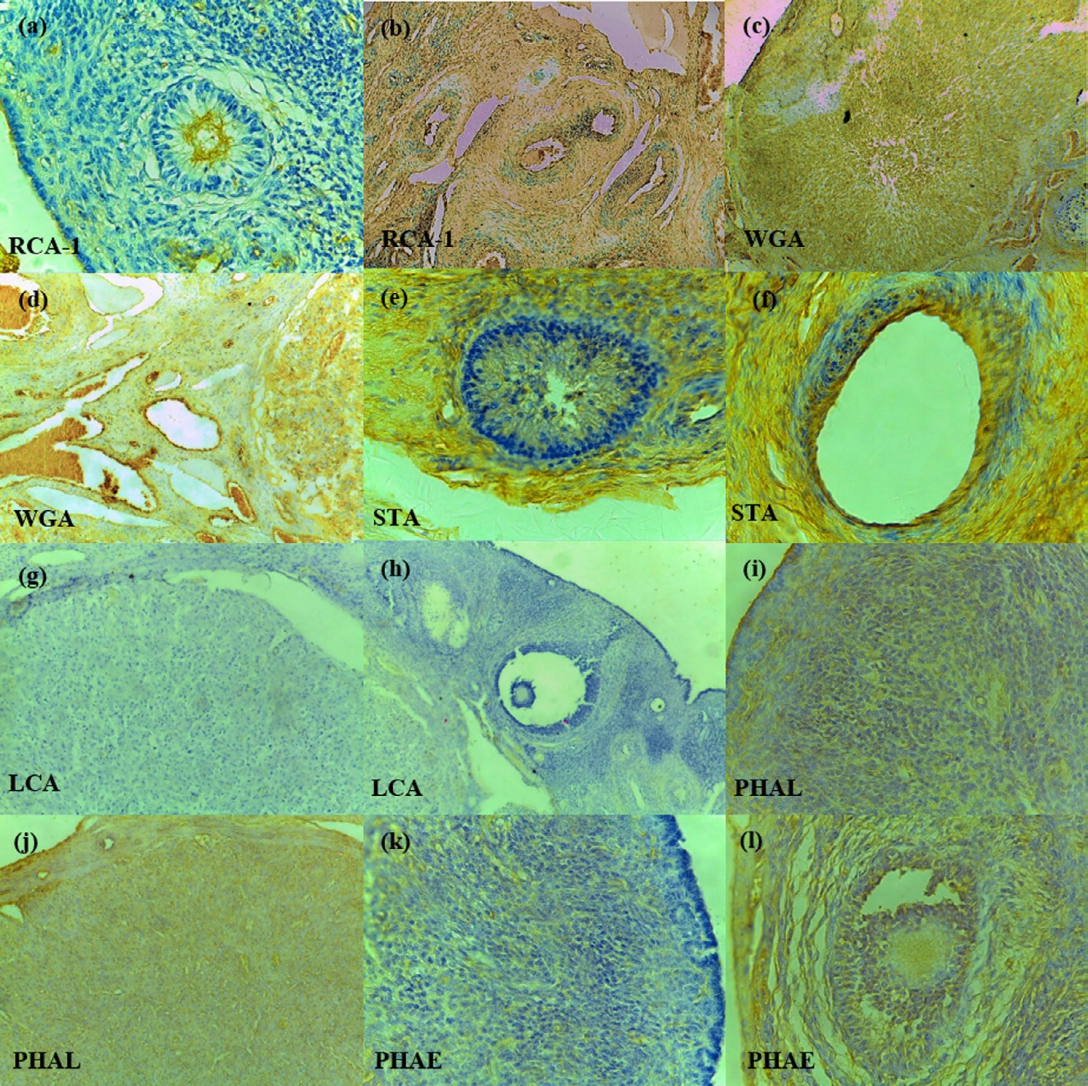
Cystic ovaries. There was a reduction of reactivity to RCA‐1 in the zona pellucida (ZP) of the primary follicle (a) (400×) and an increase in the arterial endothelium (b) (200×). Reaction to WGA was decreased in the corpus luteum (CL) (c) and increased in the vascular endothelium (d), 200×. There was a reduction of marcation to STA in ZP of secondary follicles (e) and an increase in endothelial cells (f), 400×. CL (g) and ZP of growing follicles (h) present reactivity reduced to LCA, 200×. Reaction to PHAL was reduced in mesothelial cells (i) and CL (j), 200×. PHAE is also reduced in the stroma (k) and granulosa cells of secondary follicles (l), 400×.

In cystic ovaries, changes in the reactivity of some lectins were noted in various structures of the ovarian parenchyma adjacent to the cysts. In ovaries with follicular cysts, RCA‐1 was reduced in ZP and oocyte of primordial, primary (Figure 2a) and secondary follicles, while there was a moderate increase in staining in endothelial cells (Figure 2b). With the WGA lectin, there was a reduction in CL (Figure 2c) and an increase in endothelial cells (Figure 2d). Reactivity to STA was reduced in ZP of primary and secondary follicles (Figure 2e) and increased in endothelial cells (Figure 2f). LCA was also reduced in the CL (Figure 2g) and ZP (Figure 2h) of primary and secondary follicles. With the PHA‐L lectin, reactivity was reduced in mesothelial cells (Figure 2i), CL (Figure 2j) and ZP of primary follicles, while with PHA‐E it was reduced in connective stromal cells (Figure 2k), in the oocyte of primary follicles and in the GC of secondary follicles (Figure 2l). In cases of cystic corpus luteum, the reduction in lectin staining was very similar to that observed in follicular cysts.

**FIGURE 3:**
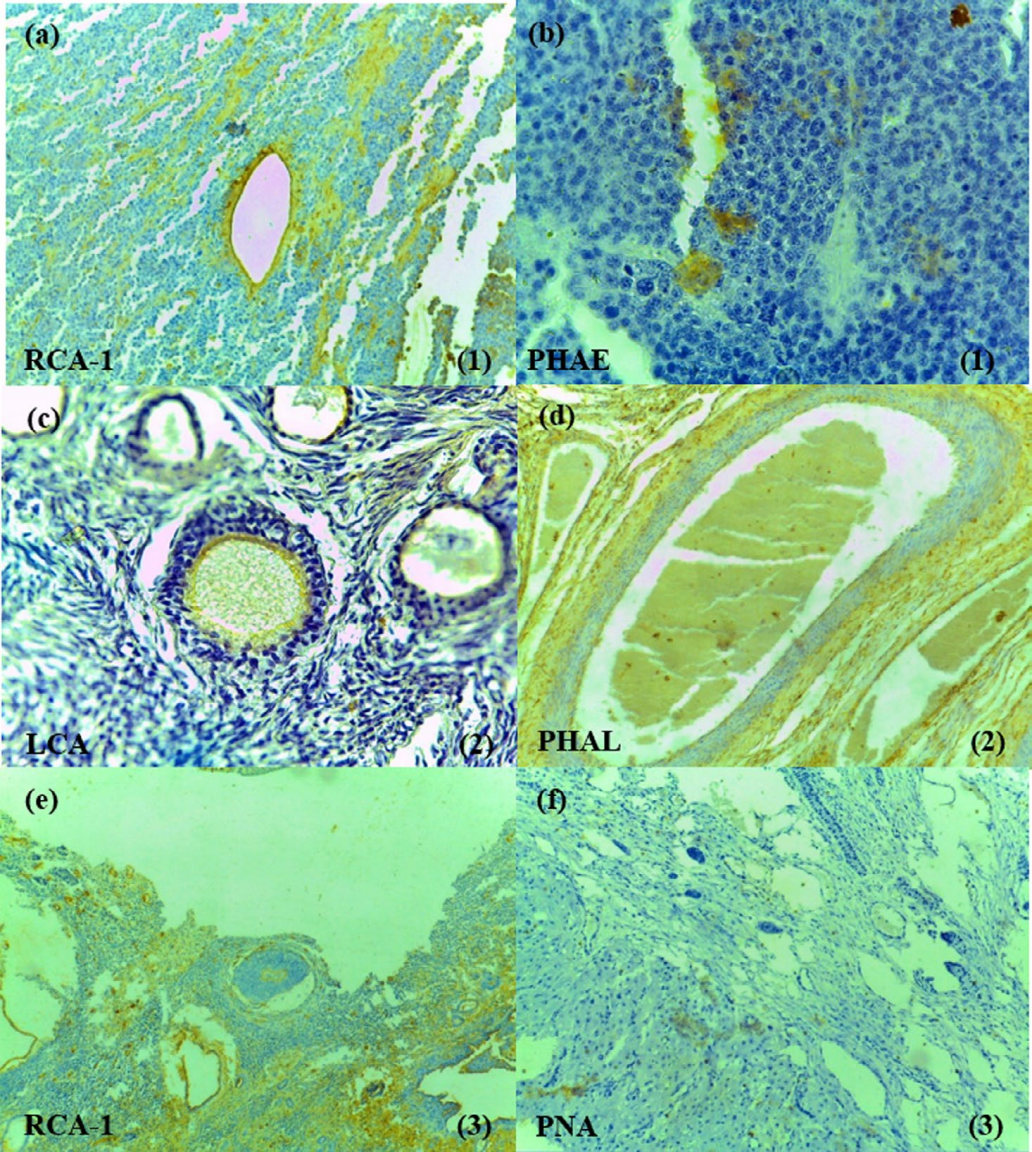
Neoplastic ovaries. Disgerminoma (1): Reduction to RCA‐1 in endothelial cells (a) and to PHAE in germ cells (b) and adjacent parenchyma was observed. Granulosa cell tumour (2): There was a reduction of reactivity to LCA in ZP of primary follicles (c) (400×) and to PHAL in vascular endothelium (d) (200×). Papillary adenocarcinoma (3): RCA‐1 and PNA marcations were reduced in ZP of pre‐antral follicles (e) and stroma (f), respectively, 200×.

Changes in the LHC pattern were also observed in neoplastic ovaries. The reaction of germ cells in dysgerminomas was usually restricted and mild to the lectins PHA‐L and PHA‐E. In the parenchyma adjacent to the neoplasm, there was a reduction in the reactivity of RCA‐1 in endothelial cells (Figure 3e) and ZP of primary and secondary follicles, of WGA in CL, of STA in CL and ZP of all follicles, of LCA in mesothelial cells, CL and ZP of primary and secondary follicles, of PHA‐L in mesothelium, CL, stroma and ZP of primary follicles, and of PHA‐E in mesothelium, ZP of primary and secondary follicles and germ cells (Figure 3f). The pattern of lectin binding in cells of granulosa cell tumour was similar to that observed in these cells of normal ovaries. In the parenchyma adjacent to this neoplasm, there was a reduction in the reactivity of WGA in oocyte of initial follicles, of STA in ZP of initial follicles, of LCA in ZP of primary (Figure 3g) and secondary follicles, of s‐WGA in PZ of primary follicles, of PHA‐L in endothelium (Figure 3h) and ZP of primary follicles, and of PHA‐E in endothelial cells and ZP of primary and secondary follicles. In cases of papillary adenocarcinoma, the neoplastic cells showed reduced staining for the lectins WGA, STA, LCA and PHA‐L, compared to the control mesothelium. In the ovarian tissue adjacent to this neoplasm, there was a reduction in reactivity to RCA‐1 in ZP of preantral follicles (Figure 3i), of PNA in connective stroma (Figure 3j), of WGA in ZP of initial follicles, of STA in ZP of all follicles, of LCA in mesothelial cells, CL and ZP of primary and secondary follicles, of s‐WGA in ZP of primary follicles, of PHA‐L in mesothelium, CL, stroma and ZP of primary follicles, and of PHA‐E in mesothelium, CG of primary and secondary follicles and ZP of secondary follicles.

## 4 Discussion

For the first time, a study characterised the binding of 14 lectins in various structures of the canine ovary, as has already been similarly demonstrated in other species, such as rodents (Salvetti et al. 2000; Barbeito et al. 2013), leporines (Parillo and Verini‐Supplizi 2001) and pigs (Mendes et al. 2019). A study carried out in canine ovaries characterised only the binding of saccharides in oocyte and ZP (Parillo and Verini‐Supplizi 1999; Parillo et al. 2005). In addition, the current study compared the lectin‐histochemical reaction in pathological ovaries (cystic and neoplastic), as previously analysed in cystic ovaries of rats (Salvetti et al. 2000; Barbeito et al. 2013) and sows (Mendes et al. 2019). Data obtained in this study are useful for understanding the cellular events and interactions that occur throughout the reproductive cycle, including the contact between gametes that occurs through interaction with the ZP. In the normal ovaries of our study, this important structure of the oocyte was positively marked mainly for the lectins RCA‐1, STA and WGA, indicating that it contains β‐D‐galactose and GlcNac residues. These data coincide with previous findings detected in ZP of bitches (Parillo and Verini‐Supplizi 1999), sows (Pastor et al. 2008; Mendes et al. 2019) and viscachas (Acuña et al. 2019). In the current study, granulosa cells of primordial and secondary follicles reacted mildly to STA, LCA, PHA‐E and PHA‐L, suggesting residues of N‐acetyl‐glucosamine, mannose, complex N‐linked sequences and glucose. These data differ from the findings in sows (Mendes et al. 2019) and are partially similar to those detected in rats (Barbeito et al. 2013). No reactivity to lectins was observed in granulosa cells of atresic follicles in our study, possibly due to the advanced degeneration process. In rats and pigs, these cells were positive to WGA and RCA‐1, even in atresia (Barbeito et al. 2013; Mendes et al. 2019). Mesothelial cells showed the highest variation and intensity of marking, mainly to WGA, STA, LCA, PHA‐L and PHA‐E, as observed in other species (Barbeito et al. 2013; Mendes et al. 2019). In the stroma of canine ovaries, a mild reaction to several lectins was noted, especially PHA‐E and PHA‐L, indicating that residues of many saccharides are deposited in the ovarian connective tissue. In addition, there was mild labeling for some lectins in blood vessels in this study; however, the endothelial cells showed subtle positivity only to STA. These data differ from those found in pigs, rats and rabbits (Parillo and Verini‐Supplizi 2001; Barbeito et al. 2013; Mendes et al. 2019). These divergent data show that these carbohydrate residues may vary in type and intensity according to the species analysed. A recent study has reported the N‐glycan pattern and N‐glycosylation sites of ZP glycoproteins in a population of porcine immature oocytes by a mass spectrometric approach (von Witzendorff et al. 2005).

Ovarian cysts are considered one of the most common reproductive lesions in several species, including bitches (Knauf et al. 2014; Arlt and Haimerl 2016). However, the mechanisms involved in the pathogenesis of this condition appear to be multifactorial and are still poorly understood (Peter 2004; Ortega et al. 2016). In animals, many investigations have demonstrated changes and mechanisms related to the etiopathogenesis of ovarian cysts, mainly in cattle (Garverick 1997; Ortega, Amable, et al. 2007; Isobe and Yoshimura 2007; Monniaux et al. 2008; Ortega et al. 2008; Rey et al. 2010; Gareis et al. 2018), rats (Anderson and Lee 1997; Ortega, Salvetti, et al. 2007; Salvetti et al. 2003; Salvetti et al. 2009) and sows (Sant'Ana, Reis Júnior, Araújo, et al. 2015; Sant'Ana, Reis Júnior, Blume, et al. 2015; Mendes et al. 2019; Grzesiak et al. 2021); however, there is little information on the genesis of the condition in canine females. The data from the present study indicate that the formation of ovarian cysts in bitches is associated with changes in the expression of carbohydrate residues in some ovarian structures. Similar studies have shown that these changes also occur in the genesis of cysts in the ovaries of sows (Mendes et al. 2019) and rats (Salvetti et al. 2003; Barbeito et al. 2013), where other structures are strongly affected, including the internal and external theca. In addition, the present study showed that the compressive effect of the two different cysts on the affected adjacent parenchyma reduced the reactivity of many lectins in various structures.

Ovarian neoplasms also represent important causes of reproductive problems in adult canine females (Sforna et al. 2003; Arlt and Haimerl 2016). In the current study, it was observed that the majority of tumour cells showed mild reactivity to few lectins. In addition, one out of three neoplasms evaluated (adenocarcinoma) showed reduced binding of lectins to neoplastic cells compared to original cells in ovaries without lesions. It is possible that this reduction occurred due to the low cell differentiation of the neoplastic cells. Similar studies have not been carried out on ovaries of domestic animals and few have analysed human ovarian neoplasms by LHC (Murakami 1991; Sasano et al. 1991). As shown in the current study, an investigation has shown that mucinous and serous ovarian cystadenomas in women contain different glycoconjugates and that the malignant transformation of tumours may be associated with alterations in these molecules (Sasano et al. 1991). Another study suggested that labeling with the lectins SBA and DBA may be useful in the differentiation and histological grade of human ovarian cystadenomas (Murakami 1991), which was not verified in the neoplasms of our bitches. Furthermore, this study revealed that the compressive effect exerted by five neoplasms compromised the reactivity of saccharides in various ovarian structures adjacent to the tumour, indicating a reduction in the physiological and endocrinological activities of the remaining ovarian tissue.

Fertilisation, an essential process that depends on glycans for sperm–oocyte recognition, may be impaired by changes in the expression of these saccharides. Glycobiological studies in mammalian reproductive biology suggest that oligosaccharides serve as functional components of glycoproteins that play critical roles in processes such as oocyte maturation, sperm–oocyte interaction, and fertilisation (El‐Mestrah and Kan 2001; Desantis et al. 2009). The zona pellucida plays a crucial role in fertilisation, and although its composition varies across species, it is generally composed of three to four acidic glycoproteins (Parillo et al. 2005). Data analysed here show that the ovary has different saccharide residues in many different structures, and that cysts and neoplasms alter the expression of these molecules. Possibly, the altered pattern of lectin binding in cystic and neoplastic ovaries occurred due to modified gene expression and enzymes that metabolise carbohydrates in these structures (Barbeito et al. 2013). The modifications in glycosidic residues may contribute to infertility in adult bitches by affecting processes such as cellular differentiation. Results of the current study suggest that fertilisation in bitches with cystic or neoplastic ovarian lesions may be compromised. In addition, our findings can contribute to expanding the understanding of the pathogenesis of some pregnancy complications such as infertility.

## Author Contributions

C.G.B. and F.J.F.S. planned the study. J.P.T., T.B.A., G.R.B., R.S.A.E., L.B.O., and F.J.F.S. collected the samples. T.B.A., F.A., L.P.O.R., and C.G.B. processed the samples for lectin histochemistry and observed the slides. F.J.F.S., J.P.T., G.R.B., R.S.A.E., F.A., and C.G.B. analysed and compared the results. J.P.T., L.B.O., and F.J.F.S. wrote the article. All the authors corrected the final version of the article.

## Conflicts of Interest

The authors declare no conflicts of interest.

## Data Availability

The data that support the findings of this study are available from the corresponding author upon reasonable request.
